# Preoperative sarcopenia and systemic immune-inflammation index can predict response to intravesical Bacillus Calmette-Guerin instillation in patients with non-muscle invasive bladder cancer

**DOI:** 10.3389/fimmu.2022.1032907

**Published:** 2022-10-07

**Authors:** Peng Liu, Shouzhen Chen, Xingzhe Gao, Hao Liang, Daqian Sun, Benkang Shi, Qiujie Zhang, Hu Guo

**Affiliations:** Department of Urology Surgery, Qilu Hospital of Shandong University, Jinan, China

**Keywords:** sarcopenia, SII, BCG - Bacille Calmette-Guérin vaccine, NMIBC (non-muscle invasive bladder cancer), immunity

## Abstract

**Background:**

To explore the prognostic significance of sarcopenia and systemic immune-inflammation index (SII) for response to intravesical Bacillus Calmette-Guerin (BCG) in patients with intermediate-, and high-risk non-muscle invasive bladder cancer (NMIBC).

**Methods:**

We retrospectively analyzed 183 consecutive patients treated in Qilu hospital of Shandong University for a first diagnosis of intermediate and high risk NMIBC. Using computed tomography scans at the third lumbar vertebra level, we calculated skeletal muscle index (SMI). Sarcopenia was defined as SMI <43 cm^2^/m^2^ for males with BMI < 25 kg/m^2^, <53 cm^2^/m^2^ for males with BMI ≥ 25 kg/m^2^, and <41 cm^2^/m^2^ for females. The response to intravesical BCG immunotherapy and relapse-free survival (RFS) were analyzed.

**Results:**

Compared with BCG responders, BCG non-responders were associated with sarcopenia (P < 0.001), carcinoma *in situ* (P < 0.001), T1 stage (P < 0.001), multiple tumor (P < 0.001), tumor diameter >=3cm (P < 0.001), and have a significant increase of neutrophil-to-lymphocyte ratio (NLR) (P < 0.001), platelet to lymphocyte ratio (PLR) (P = 0.004), SII (P < 0.001). The area under the ROC curve (AUC) of the BMI, NLR, PLR, and SII for response to intravesical BCG immunotherapy were 0.425, 0.693, 0.631, and 0.702 respectively. Logistic regression analysis demonstrated that sarcopenia and SII were predictors of response to intravesical BCG immunotherapy. The Kaplan-Meier survival analysis showed that the RFS of patients with BCG response, lower SII and no sarcopenia was significantly increased compared with that of patients with BCG non-response, higher SII and sarcopenia, respectively. Subgroup analysis demonstrated that the RFS of patients with high SII and sarcopenia was significantly decreased compared with those with low SII and no sarcopenia in Ta stage subgroup, T1 stage subgroup, non-Cis subgroup, multiple tumor subgroup, single tumor subgroup, tumor diameter≥3cm subgroup and tumor diameter<3cm subgroup, respectively (P < 0.05). However, there was no significant difference in RFS for patients in CIS subgroup (P > 0.05). Multivariate Cox analysis shown that sarcopenia (p=0.005) and high SII (*p* = 0.003) were significantly associated with poor RFS.

**Conclusions:**

Both sarcopenia and high SII are useful predictors of response to intravesical BCG in intermediate- and high-risk NMIBC patients. Patients with intermediate- and high-risk NMIBC that had sarcopenia or high SII at diagnosis were associated with poor RFS, and the combination of sarcopenia and SII may be a better predictor of RFS.

## Introduction

Bladder cancer (BC) is the 10th most commonly diagnosed cancer worldwide, and the most common genitourinary malignant tumors, with approximately 573,000 new cases and 213,000 deaths (1). It is reported that approximately 70% of patients with BC present with a disease confined to the mucosa (stage Ta and carcinoma *in situ* (CIS)) or submucosa (stage T1), also known as non-muscle invasive bladder cancer(NMIBC) (2).

The European Association of Urology (EAU) guidelines divided NMIBC patients into low-, intermediate-, and high-risk groups according to clinical features (3). The gold standard treatment for intermediate- and high-risk NMIBC is represented by transurethral resection of bladder tumor (TURBT) and a second TURB when necessary (residual tumor or absence of muscle in the specimen), followed by adjuvant intravesical treatment (3). Studies confirmed that Bacillus Calmette Guerin (BCG) is the most effective intravesical treatment for preventing the recurrence of NMIBC (4–7). Although NMIBC has a good prognosis with a 5-year overall survival rate of approximately 90%, 30–80% of cases will recur and 1–45% of cases will progress to muscle invasion within 5 years (8). In these patients, predicting BCG failure may avoid ineffective BCG therapy and its adverse effects as well as a delayed radical cystectomy (RC) procedure in some individuals. At present, the European Organization for Research and Treatment of Cancer (EORTC) model is the most commonly used tool to evaluate the recurrence and progression risk of NMIBC after intravesical BCG treatment. Although this scoring system is useful in clinical practice, the accuracy of prediction still needs to be improved (9, 10). Therefore, updating new predictors is necessary to improve risk classification and prediction of recurrence.

Nutritional status and immune function are related to occurrence and development of tumors, and muscle and inflammation status are good indicators of nutritional status and immune function. Sarcopenia is a progressive and systemic skeletal muscle disorder involving the accelerated loss of muscle mass and function that is associated with increased adverse outcomes (11). Sarcopenia is well recognized as a negative factor for immunity. Recently, accumulating evidence has suggested that sarcopenia is associated not only with a higher rate of treatment-related complications but also with a poorer oncologic prognosis, including bladder cancer (12–17). Dylan J Martini et al. (18) found that body composition may be prognostic and predictive of clinical outcome in immune checkpoint inhibitors-treated patients with urothelial carcinoma; Zhi-Bin Ke et al. (19) have proved that lower relative visceral fat area was independent predictors of response to intravesical BCG immunotherapy, suggesting that sarcopenia may be a crucial indicator of relapse after intravesical BCG treatment. Levels of inflammation are closely related to immune function. It has been reported that inflammation may play a key role in the initiation, development, and progression of several malignant tumors (20, 21). Numerous studies have demonstrated that several inflammatory markers may be associated with response to intravesical BCG immunotherapy of NMIBC, and pre-operative neutrophil-to-lymphocyte ratio (NLR) has been recommended by EUA guidelines for predicting BCG efficacy. Systemic immune-inflammation index (SII), calculated using peripheral lymphocyte, neutrophil and platelet counts, is a novel marker of inflammation level and may be related to the efficacy of BCG in patients with NMIBC. Herein, we performed a retrospective analysis to explore whether the preoperative sarcopenia and SII were associated with the response to intravesical BCG immunotherapy and prognosis in intermediate- and high-risk NMIBC patients.

## Materials and methods

### Patients

We retrospectively analyzed 183 consecutive patients treated in Qilu hospital of Shandong University for a first diagnosis of intermediate and high risk NMIBC (according to EORTC and EAU guidelines) from January 2015 to December 2021. Intravesical BCG immunotherapy consisted of one induction course (6 weekly instillations) followed by at least one year of monthly maintenance schedule. All patients accepted adequate BCG instillation and were followed up regularly. Cystoscopy and cytology were reviewed every 3 months for a period of 2 year, and every 6 months thereafter until 5 years, and then yearly (3). Random biopsies including bladder and prostatic urethra for patients were performed if necessary. The exclusion criteria are as follows (1): combined with other malignancies (2); other histological variants except urothelial carcinoma (3); active infection or hematologic neoplasms (4); incomplete data (5); previous TURBT (6); patients lost at follow-up.

### Outcomes

The primary outcome was response to intravesical BCG instillation. According to EAU guidelines (3), high grade tumor recurrence or progression to muscle invasive bladder cancer (MIBC) during follow-up were considered as BCG non-response in this study, while low grade low stage tumor recurrence should not be considered as BCG non-response. The secondary outcome was relapse-free survival (RFS), defining as the interval from the initiation of BCG therapy to the first recurrence or progression to MIBC.

### Data collect

Data were extracted from Qilu hospital of Shandong University using the hospital’s medical record system. Computed tomography (CT) images and blood parameters were collected within 7 days prior to TURBT. We selected axial images from CT scans at the third lumbar spine vertebra (L3) level when both transverse processes of L3 were visible and convert them into Digital Imaging and Communications in Medicine (DICOM) format for analysis. The delineation of skeletal muscle was performed using Slice-O-Matic software (Rev-9, Tomovision, Montreal, Quebec, Canada) and the threshold of Hounsfiled Unit (HU) was −29 to +150 HU. Skeletal muscle index (SMI) was defined as skeletal muscle area divided by patient height in meters squared. Sarcopenia was defined as SMI <43 cm^2^/m^2^ for males with BMI < 25 kg/m^2^, <53 cm^2^/m^2^ for males with BMI ≥ 25 kg/m^2^, and <41 cm^2^/m^2^ for females (22). Sarcopenia measurements were performed two times by a single radiation oncologist, and the final results were averaged. The NLR, platelet to lymphocyte ratio (PLR) and SII were used to evaluate the inflammatory status of patients. The NLR was defined as the neutrophil count divided by the lymphocyte count, the PLR as the platelets count divided by the lymphocyte count. And SII was calculated as platelet count × neutrophil count/lymphocyte count.

### Statistical analysis

Continuous variables presented as mean ± standard deviation or median (interquartile range [IQR]), categorical variables were presented as actual counts and percentages. Significance tests used to compare BCG responders with BCG non-responders were the Mann-Whitney U test or Student’s t test for continuous variables and the Chi-Square test for dichotomous parameters. Youden index refers to sensitivity+ specificity – 1 in receiver operating characteristic (ROC) curve. The optimal SII cutoff value was defined by creating a receiver operating characteristic (ROC) curve to yield the highest Youden index value. Logistic regression was used to determine independent predictors of response to intravesical BCG. We defined the outcome variable as responder. The univariate and multivariate Cox regression analyses were performed to identify independent prognostic variables affecting RFS after intravesical BCG therapy. We take <70 years, female, no smoking, no drinking, no hypertension, no diabetes, low grade, non-Cis, Ta stage, single tumor, tumor diameter< 3cm, no sarcopenia and low SII as reference. Kaplan Meier curves and log rank test were used to compare cancer free survival between the two groups. A p < 0.05 was considered statistically significant. The analyses were conducted using IBM SPSS version 25.0 (SPSS Inc., Chicago, IL, USA).

The study protocol was approved by the Ethics Committee of Qilu hospital of Shandong University. The Ethics Committee of Qilu hospital of Shandong University waived of written informed consent. The entire study was performed in accordance with the Declaration of Helsinki.

## Results

A total of 183 patients were included with a median follow-up of 30 months (IQR: 13.0–47.0). We divided the patients into two groups according to the response to intravesical BCG immunotherapy, the patients’ characteristics were shown in **Table 1**. Compared with BCG responders, BCG non-responders were associated with sarcopenia (P < 0.001), carcinoma *in situ* (P < 0.001), T1 stage (P < 0.001), multiple tumor (P < 0.001), tumor diameter >=3cm (P < 0.001), and have a significant increase of NLR (P < 0.001), PLR (P = 0.004), SII (P < 0.001).

**Table 1:** Baseline characteristics of patients stratified by response to intravesical BCG instillation.

Variables	General	BCG responders (n = 121)	BCG non-responders (n = 62)	p-value
Age (years)	62.37 ± 13.34	62.00 ± 13.20	63.06 ± 14.03	0.614
Gender				0.866
Female	40 (21.90%)	26 (21.50%)	14 (22.60%)	
Male	143 (78.10%)	95 (78.50%)	48 (77.40%)	
Smoking				0.105
Yes	71 (38.80%)	52 (43.00%)	19 (30.60%)	
No	112 (61.20%)	69 (57.00%)	43 (69.40%)	
Drinking				0.314
Yes	56 (30.60%)	40 (33.10%)	16 (25.80%)	
No	127 (69.40)	81 (66.90)	46 (74.20%)	
Hypertension				0.823
Yes	60 (32.8%)	39 (32.20%)	21 (33.90%)	
No	123 (67.20%)	82 (67.80%)	41 (66.10%)	
Diabetes				0.718
Yes	26 (14.20%)	18 (14.90%)	8 (12.90%)	
No	157 (85.8%)	103 (85.10%)	54 (87.10%)	
Grade				0.063
Low	64 (35.00%)	48 (39.70%)	16 (25.80%)	
High	119 (65.00%)	73 (60.30%)	46 (74.20%)	
Cis				<0.001
Yes	19 (10.40%)	4 (3.30%)	15 (24.20%)	
No	164 (89.60%)	117 (96.70%)	47 (75.80%)	
T stage				<0.001
Ta	73 (39.90%)	66 (54.50%)	7 (11.30%)	
T1	110 (60.1%)	55 (45.50%)	55 (88.70%)	
Tumor number				<0.001
Single	74 (40.40%)	62 (51.20%)	12 (19.40%)	
Multiple	109 (59.60%)	59 (48.80%)	50 (80.60%)	
Tumor diameter				<0.001
<3 cm	114 (62.30%)	90 (74.40%)	24 (38.70%)	
>=3 cm	69 (37.70%)	31 (25.60%)	38 (61.30%)	
Sarcopenia				<0.001
Yes	73 (39.90%)	31 (25.60%)	42 (67.70%)	
No	110 (60.10%)	90 (74.40%)	20 (32.30%)	
BMI (kg/m2)	25.06 ± 3.74	25.38 ± 3.88	24.41 ± 3.39	0.097
NLR	1.97 (1.57-2.63)	1.75 (1.39-2.44)	2.41 (1.83-3.13)	<0.001
PLR	125.31 (105.92-164.55)	120.53 (97.94-157.82)	139.10 (111.67-168.71)	0.004
SII	436.35 (321.68-657.30)	408.66 (301.23-537.93)	567.89 (408.32-814.13)	<0.001

BCG, Bacillus Calmette-Guerin; CIS, Carcinoma in Situ; BMI, Body Mass Index; NLR, Neutrophilic Lymphocyte Ratio; PLR, Platelet Lymphocyte Ratio; SII, Systemic Immune-Inflammation Index.

**Figure 1:**
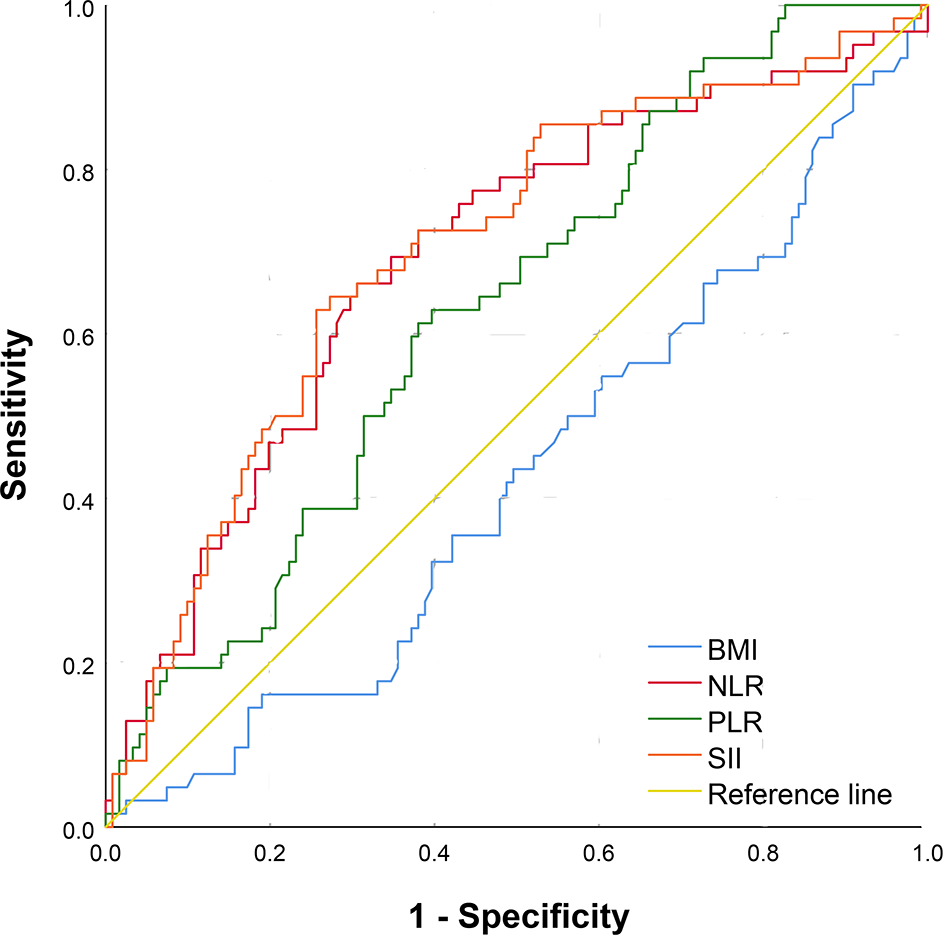
The receiver operating characteristic curve of BMI, NLR, PLR, SII in predicting response to intravesical BCG immunotherapy. BMI, Body Mass Index; NLR, Neutrophilic Lymphocyte Ratio; PLR, Platelet Lymphocyte Ratio; SII, Systemic Immune-Inflammation Index.

The area under the ROC curve (AUC) of the BMI, NLR, PLR, and SII for response to intravesical BCG immunotherapy were 0.425, 0.693, 0.631, and 0.702 respectively (**Figure 1**; **Table 2**). Therefore, BMI has limited value in predicting the response to intravesical BCG immunotherapy, whereas SII was the best immune-inflammation predictor. We use Youden index as cutoff value to divide SII into high SII group and low SII group.

**Table 2:** ROC analysis of inflammation index and BMI for response to intravesical BCG therapy.

Variables	AUC	95%CI	Youden index	Cutoff value	p-value
BMI	0.425	0.338-0.512	0.008	17.642	0.098
NLR	0.693	0.610-0.775	0.356	2.110	< 0.001
PLR	0.631	0.549-0.713	0.233	129.140	0.004
SII	0.702	0.620-0.783	0.373	514.47322	< 0.001

ROC, Receiver Operating Characteristic Curve; BMI, Body Mass Index; BCG, Bacillus Calmette-Guerin; AUC, Area under the ROC curve; NLR, Neutrophilic Lymphocyte Ratio; PLR, Platelet Lymphocyte Ratio; SII, Systemic Immune-Inflammation Index.

**Figure 2:**
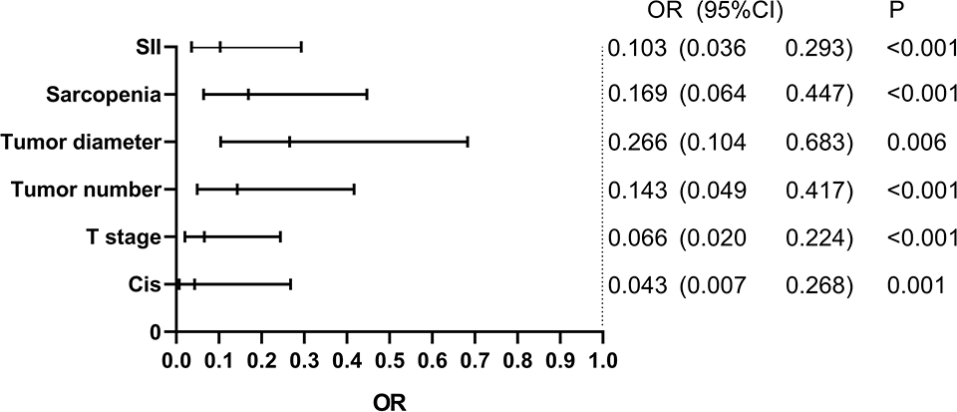
Multivariate logistic regression analysis for response to BCG. BCG, Bacillus Calmette-Guerin; SII, Systemic Immune-Inflammation Index; CIS, Carcinoma in Situ.

In univariable logistic regression analysis, seven factors (grade, T stage, CIS, tumor number, tumor diameter, SII, and sarcopenia) reached statistical significance. Then, above seven factors were included in the multivariate logistic analysis. The variables with p <0.05 were identified as independent predictors of response to intravesical BCG, including T stage, Cis, tumor number, tumor diameter, SII, and sarcopenia (**Figure 2**).

**Figure 3:**
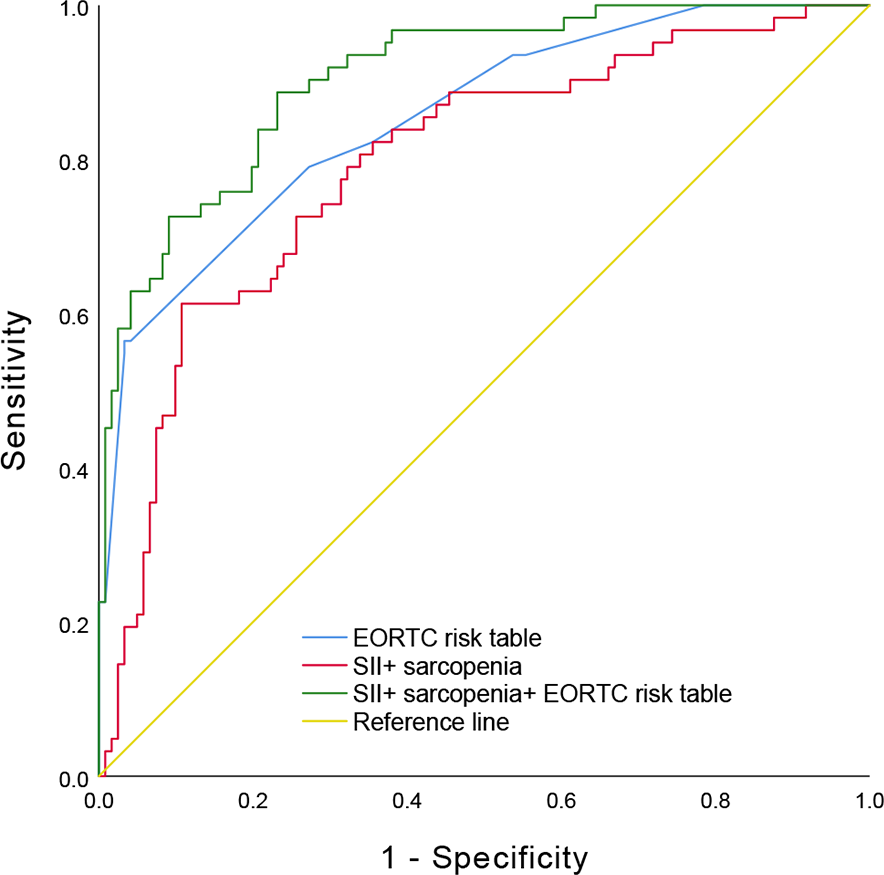
The receiver operating characteristic curve comparing preoperative sarcopenia and SII with EORTC risk table. SII, Systemic Immune-Inflammation Index; EORTC, European Organization for Research and Treatment of Cancer.

To explore the prognostic value of combined preoperative sarcopenia with SII for response to intravesical BCG immunotherapy in NMIBC, ROC curve comparing preoperative sarcopenia and SII with EORTC risk table was performed. The area under the ROC curve of the preoperative sarcopenia and SII, EORTC risk table, preoperative sarcopenia and SII + EORTC risk table for response to intravesical BCG immunotherapy were 0.793, 0.853, and 0.906 respectively (**Figure 3**).

**Figure 4:**
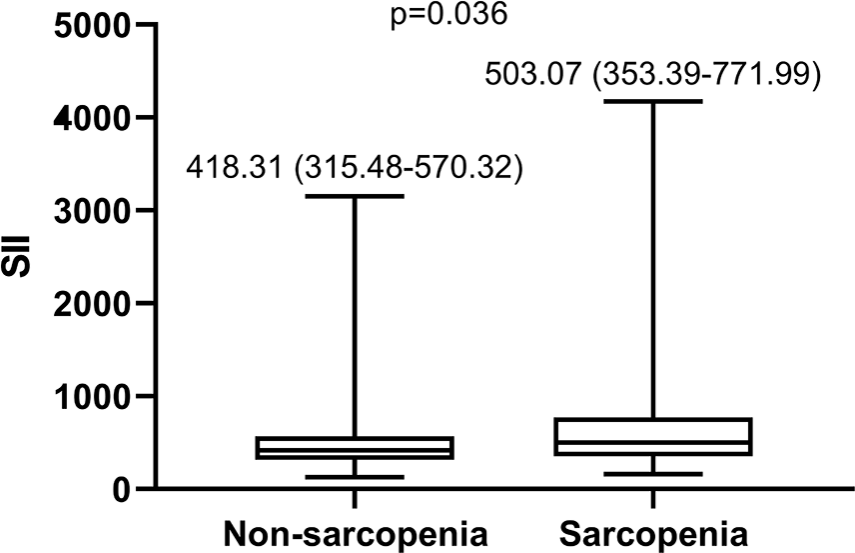
Boxplot of SII in patients with sarcopenia and without sarcopeni. SII, Systemic Immune-Inflammation Index.

Boxplot (**Figure 4**) showed that patients with sarcopenia had significantly higher SII value than patients without sarcopenia (503.07 (353.39- 771.99) versus 418.31 (315.48- 570.32), p= 0.036).

**Figure 5:**
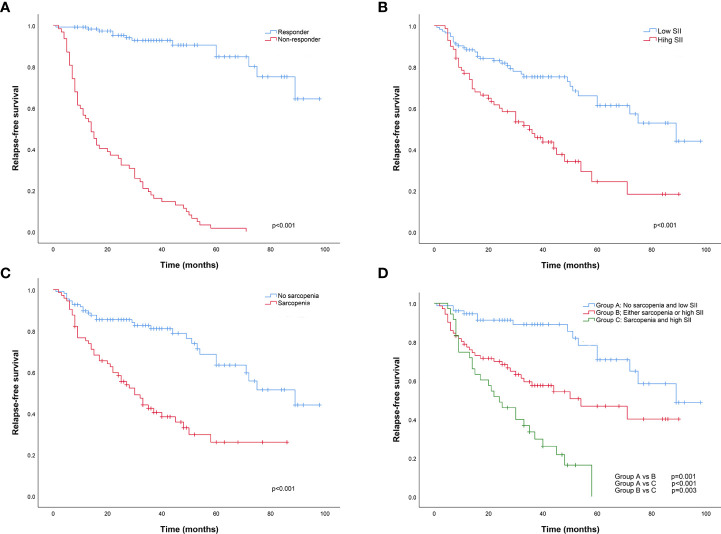
The Kaplan-Meier relapse-free survival analysis between BCG responder and non-responder **(A)**, low and high SII group **(B)**, no sarcopenia and sarcopenia group **(C)**, No sarcopenia and low SII group, Either sarcopenia or high SII group, and Sarcopenia and high SII group **(D)**, respectively. BCG, Bacillus Calmette-Guerin; SII, Systemic Immune-Inflammation Index.

**Figure 6:**
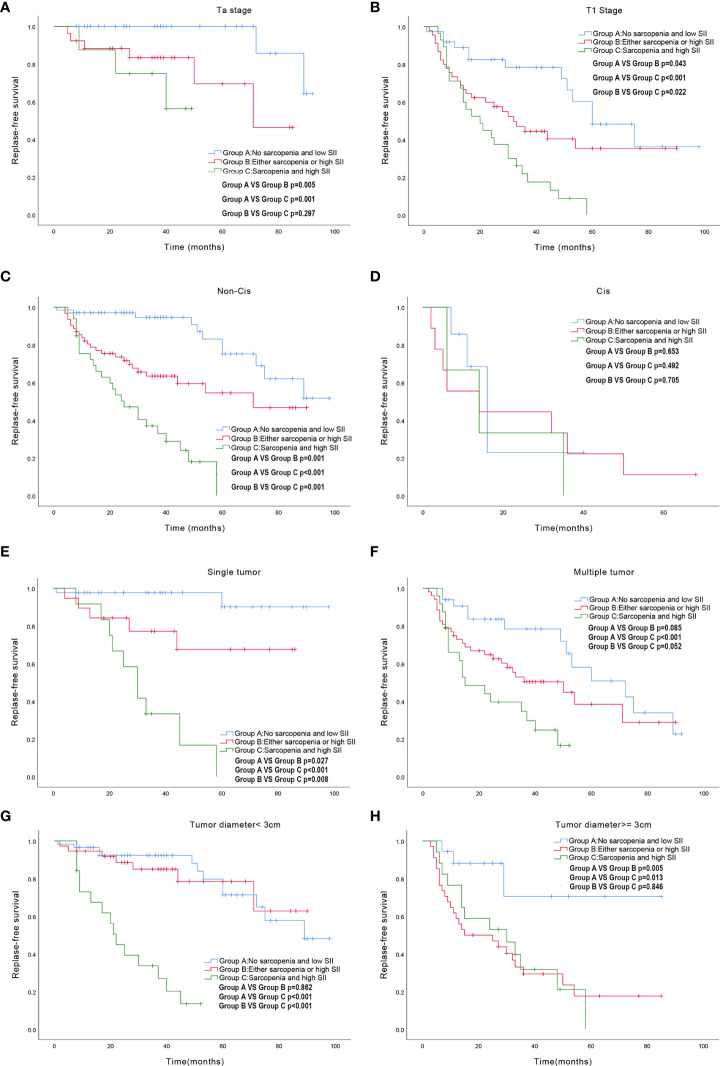
Subgroup survival analysis exploring the effect of sarcopenia accompanied by high SII on RFS. **(A)** Ta stage subgroup; **(B)** T1 stage subgroup; **(C)** Non-CIS subgroup; **(D)**CIS subgroup; **(E)** single tumor subgroup; **(F)** multiple tumor subgroup; **(G)** tumor diameter<3cm subgroup; **(H)** tumor diameter>=3cm subgroup. SII: Systemic Immune-Inflammation Index; RFS: Relapse-free survival; CIS: Carcinoma in Situ.

The Kaplan-Meier survival analysis showed that the RFS of patients with BCG response, lower SII and no sarcopenia was significantly increased compared with that of patients with BCG non-response, higher SII and sarcopenia, respectively (**Figure 5**). Based on this result, we analyzed survival depending on sarcopenia and SII. Patients were divided into three groups according to sarcopenia and SII: patients with no sarcopenia and low SII, patients with sarcopenia and high SII, and patients with either sarcopenia or high SII. The survival of patients exhibiting sarcopenia accompanied by high SII was significantly poorer than those with no sarcopenia and low SII (**Figure 5**). Further, we performed subgroup analysis of the above results according to T stage, CIS, tumor number, tumor diameter. Subgroup analysis demonstrated that the RFS of patients with high SII and sarcopenia was significantly decreased compared with those with low SII and no sarcopenia in Ta stage subgroup, T1 stage subgroup, non-Cis subgroup, multiple tumor subgroup, single tumor subgroup, tumor diameter≥3cm subgroup and tumor diameter<3cm subgroup, respectively (P < 0.05) (**Figure 6**). However, there was no significant difference in RFS for patients in CIS subgroup (P > 0.05). Therefore, sarcopenia accompanied by high SII has limited predictive value on RFS in NMIBC patients with CIS (**Figure 6**).

The results of univariate and multivariate Cox analysis were summarized in **Table 3**. In univariate Cox analysis, seven variables were associated with survival (p< 0.1), including smoking, grade, Cis, T stage, tumor number, tumor diameter, sarcopenia and SII, and those factors were included into multivariate Cox analysis. In the multivariate Cox analysis, group with high grade (p=0.015), CIS (p=0.030), T1 stage (p=0.001), multiple tumor (p<0.001), tumor diameter≥3cm (p=0.036), sarcopenia (p=0.005) and high SII (*p* = 0.003) were significantly associated with poor RFS.

**Table 3:** Univariable and multivariable cox analyses of RFS.

Subject characteristics	Univariate	Multivariate		
	HR (95% CI)	P value	HR (95% CI)	P value
Age (<70 years, >=70 years)	1.190 (0.745-1.923)	0.468		
Sex (female, male)	1.132 (0.656-1.949)	0.667		
Smoking (no, yes)	1.560 (0.942-2.525)	0.085	0.862 (0.515-1.449)	0.566
Drinking (no, yes)	0.744 (0.445-1.259)	0.275		
Hypertension (no, yes)	1.226 (0.747-2.036)	0.410		
Diabetes (no, yes)	1.038 (0.529-2.028)	0.923		
Grade (low, high)	2.164 (1.280-3.690)	0.004	2.040 (1.149-3.371)	0.015
CIS (no, yes)	3.773 (2.105-7.142)	<0.001	2.040 (1.075-3.571)	0.030
T stage (Ta, T1)	4.739 (2.463-8.849)	<0.001	3.030 (1.666-6.666)	0.001
Tumor number (single, multiple)	2.816 (1.639-5.00)	<0.001	3.125 (1.754-5.555)	< 0.001
Tumor diameter (<3cm, ≥3cm)	3.030 (1.886-4.901)	<0.001	1.785 (1.334-3.030)	0.036
Sarcopenia (no, yes)	3.225 (1.960-5.263)	<0.001	2.040 (1.250-3.333)	0.005
SII (low, high)	2.631 (1.666-4.166)	<0.001	2.127 (1.298-3.571)	0.003

RFS, relapse-free survival; CIS, Carcinoma in Situ; BMI, Body Mass Index; SII, Systemic Immune-Inflammation Index.

## Discussion

The results of the present study demonstrated that sarcopenia and SII were associated with response to intravesical BCG treatment and RFS in intermediate- and high-risk NMIBC. We conducted a ROC curve comparing preoperative sarcopenia and SII with EORTC risk table to explore the prognostic value of combined preoperative sarcopenia with SII for response to intravesical BCG immunotherapy in NMIBC. The area under the ROC curve proves that preoperative sarcopenia combined with SII has significant prediction value for response to intravesical BCG immunotherapy in NMIBC, and combining it with EORTC risk table can improve the prediction accuracy of EORTC risk table. Furthermore, we divided the patients into three groups: sarcopenia and high SII, either sarcopenia or high SII, no sarcopenia and low SII. And, we interestingly found that patients with sarcopenia and high SII showed the poorest RFS, and patients with no sarcopenia and low SII had the best RFS, suggesting that sarcopenia accompanied by high SII was a valuable predictor of poor RFS in those patients.

Sarcopenia, defined as progressive loss of muscle mass and function, is associated with poor nutritional status and immune function, while poor nutritional status and immune function may lead to further decreases in physical capacity and progression of disease. In this study, CT scans at L3 level were used to assess sarcopenia, and sarcopenia was defined as SMI <43 cm^2^/m^2^ for males with BMI < 25 kg/m^2^, <53 cm^2^/m^2^ for males with BMI ≥ 25 kg/m^2^, and <41 cm^2^/m^2^ for females. The interaction between sarcopenia and cancer has been the focus of research in recent years, and a large number of studies have proved that sarcopenia often predicts poor survival outcomes in tumor patients (12, 13). Besides, Zhi-Bin Ke et al. also demonstrated that lower relative visceral fat area was vital independent predictors of response to intravesical BCG immunotherapy and was associated with preferable prognosis in NMIBC patients (19). This is the first study to explore the relationship between sarcopenia and response to intravesical BCG instillation. The mechanisms underlying the prognostic value of sarcopenia in response to intravesical BCG instillation may include the following two points. First, sarcopenia often occurs in the elderly and often represents poor nutritional status, so their immune function and response to BCG are poor. In addition, skeletal muscle is no longer considered as a pure motor unit, but more and more considered as an organ with immune regulatory properties in the past two decades. IL-15 is a muscle derived myokine that can regulate the proliferation and activation of natural killer cells, CD8 + T cells, and B-cells in the circulation to enhance immunity (23). More importantly, Emma Kurz et al. identifies an important role for exercise in driving an immune-mediated anti-tumor effect in pancreatic cancer through the activation of the IL-15/IL-15Rα axis (24). Therefore, lower levels of some myokines in patients with sarcopenia may affect the efficacy of intravesical BCG instillation. Third, studies have shown that the mechanism of BCG is mainly the increase of T lymphocytes, with a predominance of T helper/inducer cells. Cytokines associated with the development of sarcopenia, such as transforming growth factor (TNF)-β (25) and interleukin (IL)-6 (26), cause T-cell exhaustion, leading to BCG failure in NMIBC patients. Taking sarcopenia as one of the predictors of response to intravesical BCG can improve the accuracy of prediction and select patients with poor intravesical BCG effect as soon as possible for follow-up treatment, so as to avoid recurrence or progression to MIBC.

We also proved that SII value preoperatively evaluated could be a valuable tool to predict BCG response in patients with NMIBC. The results of this study showed that NLR, PLR and SII of BCG non-responders were significantly higher than those of BCG responders. ROC curve revealed that SII was superior to NLR or PLR in predicting the response to intravesical BCG treatment. We also proved that SII value preoperatively evaluated was a valuable tool to predict BCG response and an independent prognostic factor for RFS after BCG therapy in intermediate- and high- risk NMIBC patients. Moreover, we proved that SII combined with sarcopenia can predict response to intravesical BCG immunotherapy and RFS in intermediate- and high- risk NMIBC patients.

In recent years, more and more attention has been paid to the relationship between systemic inflammation and muscle consumption (27, 28). Interestingly, both sarcopenia and inflammation are common in the elderly, who are prone to bladder cancer. Persistent inflammation may contribute to sarcopenia (29). The inflammatory response would have consumed energy and proinflammatory cytokines such as IL-6, TNF-α could lead to muscle damage (30). Moreover, the dysfunction of skeletal muscle tissue can release TNF, TWEAK and IL-6, leading to chronic inflammatory response (31). In this study, we also found that patients with sarcopenia had significantly higher SII value than patients without sarcopenia. The mechanisms underlying the inflammatory response and sarcopenia during NMIBC may lead to new treatments to prevent sarcopenia in NMIBC patients, and may find novel methods to treat bladder cancer. Furthermore, sarcopenia and inflammation are closely related and promote each other in NMIBC patients (32), and they all damage the immune function. Therefore, we believe that SII accompanied by sarcopenia has significant value in predicting response to BCG in NMIBC patients.

There are some limitations in this study. First, results should be interpreted with caution due to its retrospective nature and limited sample size. Second, although CT is the gold standard imaging modalities for assessing muscle mass and quality, there is no clear cutoff value to identify sarcopenia. In this study, we used criterion defined by Martin et al. (22), which has been used to define sarcopenia in most previous studies on bladder cancer (33). Third, this study only included the Chinese population and did not represent other ethnic groups. Despite these limitations, this is the first study to explore that sarcopenia and SII are predictors of response to intravesical BCG in intermediate- and high- risk NMIBC patients.

## Conclusion

Both sarcopenia and high SII were useful predictors of response to intravesical BCG in intermediate- and high-risk NMIBC patients. Patients with intermediate- and high-risk NMIBC that had sarcopenia or high SII at diagnosis were associated with poor RFS, and the combination of sarcopenia and SII may be a better predictor of RFS.

## Data availability statement

The raw data supporting the conclusions of this article will be made available by the authors, without undue reservation.

## Ethics statement

The studies involving human participants were reviewed and approved by Medical Ethics Committee of Qilu Hospital of Shandong University. Written informed consent for participation was not required for this study in accordance with the national legislation and the institutional requirements.

## Author contributions

Protocol development: PL, SC, XG, HL, DS, BS, QZ, HG. Data collection and management: PL, DS Data analysis: PL, SC, XG, HL. Manuscript writing: PL, SC, QZ, HG. All authors contributed to the article and approved the submitted version.

## Funding

Shandong Medical and Health Science and Technology Development Plan Project (2018WS333), the National Natural Science Foundation of China (Grant No. 81970661; 81900637) and the Tai Shan Scholar Foundation to BS (ts201511092).

## Conflict of interest

The authors declare that the research was conducted in the absence of any commercial or financial relationships that could be construed as a potential conflict of interest.

## Publisher’s note

All claims expressed in this article are solely those of the authors and do not necessarily represent those of their affiliated organizations, or those of the publisher, the editors and the reviewers. Any product that may be evaluated in this article, or claim that may be made by its manufacturer, is not guaranteed or endorsed by the publisher.
